# Chemical and flavour dynamics in *Cyperus esculentus* L. pomace-raspberry composite fruit wine fermentation: A combined UHPLC-OE-MS and HS-SPME-GC–MS approach

**DOI:** 10.1016/j.fochx.2025.103234

**Published:** 2025-11-03

**Authors:** Chang Yu, Yang Liu, Yintu Na, Xiaotong Wu

**Affiliations:** a Institution: Inner Mongolia University, Address: Inner Mongolia University, located at Hohhot City, Inner Mongolia Autonomous Region, China; b Institution: Inner Mongolia Traditional Chinese and Mongolian Medical Research Institute, No. 1, 35 Middle West Lane, Saihan District, Hohhot, Inner Mongolia Autonomous Region, China

**Keywords:** *Cyperus esculentus* meal, Red raspberry, Fruit wines, Volatile components, Chemical and flavour profiles

## Abstract

To increase the flavour complexity and overall quality of pure raspberry fruit wine, a novel composite fruit wine was developed using *Cyperus esculentus* meal saccharification liquid and red raspberries as raw materials. In this study, ultrahigh-performance liquid chromatography with online extraction and mass spectrometry (UHPLC-OE-MS), as well as headspace solid-phase microextraction coupled with gas chromatography–mass spectrometry (HS–SPME–GC–MS), were used to investigate the impact of raspberry saccharification liquid on the chemical composition and flavour profile of the composite fruit wine. The findings are summarized as follows:

Significant differences were observed in the composition of sugars, amino acids, and fatty acids between the composite fruit wine and pure raspberry wine. Notable increases were detected in nonanedioic acid, chlorogenic acid, D-tartaric acid, and gluconic acid. Additionally, substantial increases were detected in the levels of esters, terpenes, alcohols, acids, aldehydes, and ketones, including unique compounds such as decanoic acid ethyl ester, 2,3-dehydro-1,8-eucalyptol, and absynthin, as well as cyclohexanol,1-methyl-4-(1-methylbutyl)-trans-1,5-heptadiene-4-one, and 3,3,6-trimethylhexanoic acid. In contrast, the concentrations of compounds such as succinic acid, propanal, 4-hydroxyproline, and ethyl acetate significantly decreased.

This modification increased various aromatic compounds, enriching the flavour profile of the fruit wine and enhancing its complexity and overall aromatic characteristics. The findings of this study provide valuable insights for the development of high-quality composite fruit wines.

## Highlights

• Cyperus esculentus meal saccharification elevates flavor and nutritional value of raspberry fruit wine.
• GC–MS method was used to identify aroma compounds in compound fruit wines.
• Analysis of differential metabolites in complex fruit wines by UHPLC-OE-MS method.

## 1 Introduction

Red raspberries are berries with high nutritional value and are rich in various functional components and nutrients. Red raspberry wine boasts a distinctive flavour profile and is highly favoured by consumers. Its quality and taste are influenced by fermentation substrate factors (Jing et al., 2022;Satir, 2022). However, traditional fermentation of pure red raspberry wine has limitations, resulting in deficiencies in mouthfeel, flavour, and quality. For instance, while red raspberry wine has intense fruit aromas, it typically has an overly acidic profile (Huang et al., 2023). Its bitterness and tannic astringency contribute to inferior quality (Chen et al., 2021), whereas the higher alcohols produced during fermentation mask its ideal fruit characteristics, leading to flavour disharmony (Wang et al., 2025). Previous studies analysing the saccharification broth components of jatropha have revealed its low protein content, high starch content, and distinctive flavour profile, which includes nutrients such as sterols, alkaloids, tannins, and flavonoids (Wu et al., 2022;Tian et al., 2023). During fermentation, proteins in jatropha powder are converted by yeast into amino acids (Coelho et al., 2018). Moreover, the saccharified liquid provides an abundant carbon source for microorganisms.

Therefore, this study proposes cofermenting saccharified *Cyperus esculentus* meal liquid with red raspberries. To date, no studies have conducted qualitative and quantitative analyses of the primary components in such mixed-fermentation fruit wines. Saccharified liquid contains diverse nutrients; incorporating it as a fermentation substrate into conventional raspberry wine introduces these rich nutrients into the final product. This supplementation may not only alter the nonvolatile components of the wine but also influence its flavour characteristics, thereby enhancing overall quality.

In this study, UHPLC-OE-MS and HS-SPME-GC–MS were used to compare the aroma profiles and compound concentrations between *C. esculentus* meal raspberry wine and traditional raspberry wine. The results indicate that red raspberry wine supplemented with *C. esculentus* saccharification liquid contains the resveratrol analogue pterostilbene—a potent antioxidant offering cardiovascular protection—alongside glutathione (a natural antioxidant) and D-tartaric acid (a natural antimicrobial). These components may synergistically enhance the wine's flavour and functional properties. This study of cofermentation between *Cyperus esculentus* and raspberries aims to improve fruit wine flavour, reduce fermentation costs, and investigate the effects of saccharified liquid on yeast metabolic pathways during fermentation. This reveals the mechanism by which yeast utilizes fermentation substrates to promote flavour formation.

## 2 Materials and methods

### 2.1 Experimental materials

The saccharified solution of *C. esculentus* pomace was provided by the School of Life Sciences at Inner Mongolia University. It was directly used in subsequent fermentation experiments, and the *Rubus idaeus* L. Heritage variety was crushed and stirred in a juicer to prepare a pulp.

*C. esculentus* saccharification liquid and red raspberry fruit pulp were mixed proportionally. To kill bacteria and wild yeast and ensure the purity and flavour of the fruit wine, the mixture was placed in an 80 °C water bath for 15 min and then cooled to room temperature.

### 2.2 Yeast activation and culture

A total of 1.5 g of dry Angel yeast (Angel Yeast Co. Ltd.) was dissolved in 100 mL of glucose solution, activated at 30 °C for 30 min, transferred to YPD liquid medium, and cultured for 19 h to obtain the yeast liquid. The yeast concentration was approximately 4 × 10^7^ CFU/mL.

Lalvin 71B yeast (Lallemand) was cultured in the same way as Angel yeast, with a yeast concentration of approximately 2 × 10^7^ CFU/mL.

### 2.3 Experimental design and fermentation process

In accordance with the optimal processing parameters determined by our team's previous pre-experiment, the sugar level of the fruit wines was adjusted to 18°Brix. After inoculation with a 3 % (*v*/v) yeast suspension, the fermentation substrate with a raw material ratio of 1:2 was left to ferment at 28 °C for 7 days. Analysis of the alcohol content in the resulting compound fruit wine yielded a measured value of 7.6 % (*v*/v). The texture was uniform and free of impurities.

This experiment included two treatment groups: Group D was a mixture of raspberry fruit pulp and *C. esculentus* saccharification liquid, and Group S was a raspberry fruit pulp-only fermentation group. The treatment was repeated on each group 6 times.

### 2.4 Chemicals

All the reagents used were of analytical grade (purity >95 %), and all the solvents were of HPLC grade (purity >98 %). Methanol and acetonitrile were acquired from CNW Technologies. Ammonium acetate was purchased from SIGMA-ALDRICH. Ethanoic acid was purchased from Fisher Chemical. Ultrapure water was obtained from Watsons.

### 2.5 UHPLC-OE-MS analysis

#### 2.5.1 Metabolite extraction

One hundred microlitres of the sample was transferred to an EP tube. After the addition of 400 μL of extract solution (methanol, containing an isotopically labelled internal standard mixture), the samples were vortexed for 30 s, sonicated for 10 min in an ice–water bath, and incubated for 1 h at −40 °C to precipitate the proteins. Afterwards, the sample was centrifuged at 12000 rpm (RCF = 13,800 ×g; *R* = 8.6 cm) for 15 min at 4 °C. The resulting supernatant was transferred to a fresh glass vial for analysis. The quality control (QC) sample was prepared by mixing equal amounts of the supernatants from all of the samples.

UHPLC-OE-MS analyses were performed using a UHPLC system (Vanquish, Thermo Fisher Scientific) with a UPLC HSS T3 column (2.1 mm × 100 mm, 1.8 μm) coupled to an Orbitrap Exploris 120 mass spectrometer (Orbitrap MS, Thermo). The mobile phase consisted of 5 mmol/L ammonium acetate and 5 mmol/L acetic acid in water (A) and acetonitrile (B). The autosampler temperature was 4 °C, and the injection volume was 2 μL. An Orbitrap Exploris 120 mass spectrometer was used because of its ability to acquire MS/MS spectra in information-dependent acquisition (IDA) mode in the control of the acquisition software (Xcalibur, Thermo). In this mode, the acquisition software continuously evaluates the full-scan MS spectrum. The ESI source conditions were set as follows: sheath gas flow rate of 50 Arb, aux gas flow rate of 15 Arb, capillary temperature of 320 °C, full MS resolution of 60,000, MS/MS resolution of 15,000 collision energy of 10/30/60 in NCE mode, and spray voltage of 3.8 kV (positive) or − 3.4 kV (negative).

#### 2.5.2 Data preprocessing and annotation

The raw data were converted to the mzXML format using ProteoWizard and processed with an in-house program, which was developed using R and based on XCMS, for peak detection, extraction, alignment, and integration. An in-house MS2 database (BiotreeDB) was subsequently used for metabolite annotation. The cut-off for annotation was set at 0.3.

### 2.6 Solid-phase microextraction

The samples were removed from the refrigerator at −80 °C for liquid nitrogen grinding, vortexed to mix well, and approximately 500 mg (1 mL of liquid) of each sample was weighed into a headspace vial; saturated NaCl solution was added, and 20 μL (10 μg/mL) of the internal standard solution was added; the samples were extracted by fully automated headspace solid-phase microextraction (HS–SPME) for GC–MS analysis.

### 2.7 GC–MS analysis

The sample was shaken at a constant temperature of 60 °C for 5 min, and the 120 μm DVB/CWR/PDMS extractor head was inserted into the headspace vial of the sample. Headspace extraction was carried out for 15 min. The sample was resolved at 250 °C for 5 min, and then separated and identified by GC–MS. The extractor head was aged in a Fibre Conditioning Station at 250 °C for 5 min before sampling.

A DB-5MS capillary column (30 m × 0.25 mm × 0.25 μm, Agilent J&W Scientific, Folsom, CA, USA) with high-purity helium (purity not less than 99.999 %) as the carrier gas, a constant flow rate of 1.2 mL/min, an inlet temperature of 250 °C, non-split injection, and a solvent delay of 3.5 min was used. The temperature program was as follows: 40 °C for 3.5 min, 10 °C/min to 100 °C, then 7 °C/min to 180 °C, and finally 25 °C/min to 280 °C for 5 min.

An electron bombardment ion source (EI), an ion source temperature 230 °C, a four-stage rod temperature of 150 °C, a mass spectrometry interface temperature of 280 °C, an electron energy of 70 eV, scanning in ion detection mode (SIM), and accurate qualitative and quantitative ion scanning (GB 23200.8–2016) were used.

#### 2.7.1 Data preprocessing and annotation

The principles of qualitative and quantitative metabolites are based on multiple species, the literature, some standards, and retention indices, and a database is independently established, including the determined RT and qualitative and quantitative ions, for selecting the ion detection mode for accurate scanning. One quantitative ion and 2–3 qualitative ions are selected for each compound. All ions to be detected in each group were detected in time segments according to the peak order. If the detected retention time is consistent with the standard reference and if all the selected ions appear in the sample quality spectrum after the background is subtracted, the substance will be determined as the material. Quantitative ions are selected for integration and calibration to increase the accuracy of the quantitative work.

## 3 Results and discussion

### 3.1 Metabolomics analysis reveals that sugar syrup from *C. esculentus* reshapes the metabolic profile of raspberry wine

To obtain a comprehensive understanding of the differences in metabolites of red raspberry fruit wine at the end of fermentation with the addition of *C. esculentus* saccharification liquid, nontargeted metabolomics analysis of the two wines was carried out using the UHPLC–OE–MS method; 569 substances were detected by MS/MS, and 211 active substances were screened out on the basis of comparisons with the Human Metabolome Database (HMDB) and the Kyoto Encyclopedia of Genes and Genomes (KEGG).

**Fig. 1:**
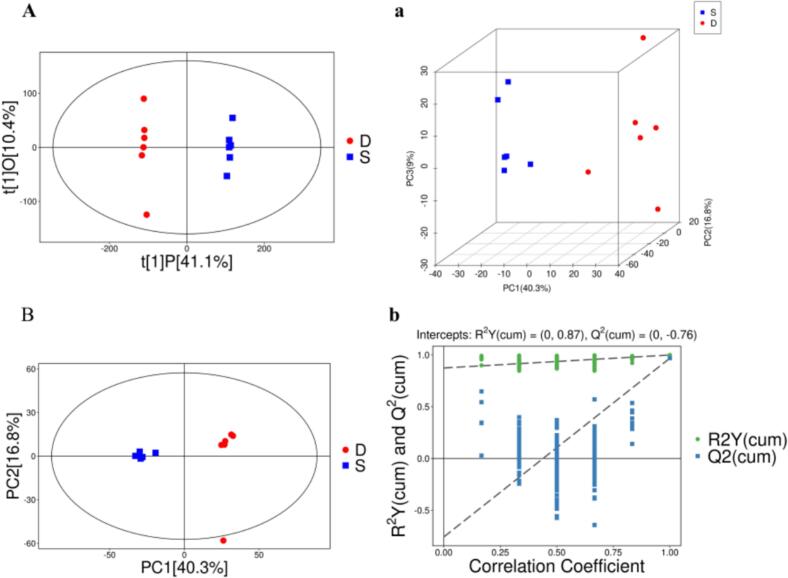
A UHPLC-OE-MS metabonomic analysis of two fruit wines. (A) Score plot (PCA) for two group wines. (a) Score plot 3D (PCA) for two group wines. (B)OPLS-DA Score plot for two group wines. (b) OPLS-DA permutation plot for two group wines.

As shown in Fig. 1A and a, principal component analysis clearly distinguished the samples (PC1 + PC2: 57.1 %; PC1: 40.3 %, PC2: 16.8 %), confirming the metabolic profile differences among the wine samples. The subsequent OPLS-DA model employed in further analysis proved effective without overfitting (R^2^Y(cum) = 0.87; Q^2^(cum) = −0.076), demonstrating robust predictive capability. (Fig. 1B and b). The addition of oilseed sugar solution was confirmed to significantly alter the metabolic profile of raspberry wine.

**Fig. 2:**
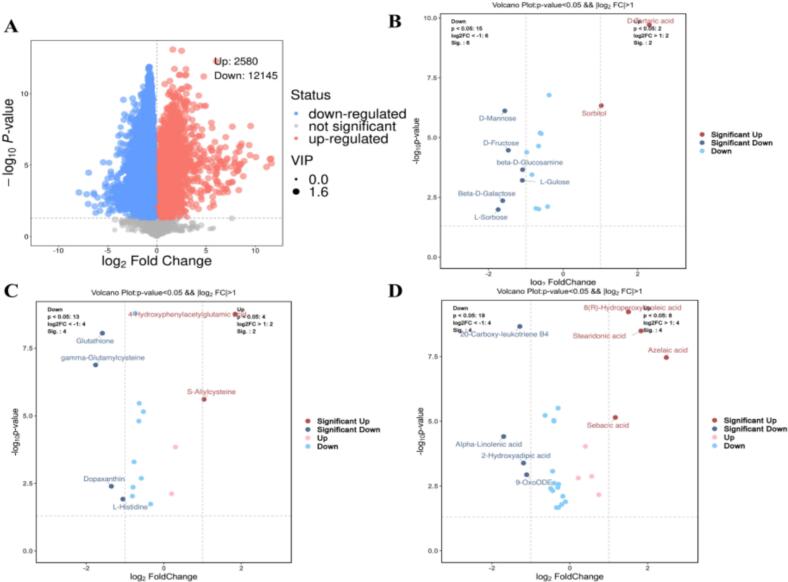
A UHPLC-OE-MS metabonomic analysis of two fruit wines.

As shown in Fig. 2, metabolites were selected on the basis of the combined criteria of variable importance projection (VIP) values >1.6 derived from the OPLS-DA model, *p* values <0.05 from Student's *t*-tests, and |log2FC| > 1 (fold change >2). Only metabolites satisfying all three criteria were identified as significantly different and are highlighted in the volcano plot.

A total of 17 sugar compounds were detected in the fruit wines (Fig. 2B), and the contents of sorbitol and D-tartaric acid significantly increased, whereas the contents of L-sorbose, L-gulose, d-fructose, D-mannose, β-D-galactose, and β-D-glucosamine significantly decreased. The changes in polysaccharide composition not only stem from differences in raw material components but also may be related to yeast growth and metabolic processes. Polysaccharides significantly influence multiple process characteristics and quality attributes of wine. The specific consumption of fermentable sugars (such as d-fructose and D-mannose) indicates enhanced glycolytic flux, driving the production of carbon skeletons (e.g., pyruvate) and energy required for subsequent flavour compound biosynthesis.

Simultaneously, 17 amino acids and their derivatives were identified. We observed significant decreases in the levels of glutathione, glutathione synthase, dopachrome, and L-histidine and increases in the levels of allyl cysteine and *N*-phenylacetyl-l-glutamine (Fig. 2C). The presence of amino acids provided a relatively abundant nitrogen source for the fermentation process, effectively shortening the fermentation cycle (Wang et al., 2021). More importantly, the consumption of amino acids such as L-histidine holds particular mechanistic significance. During fermentation, yeast converts nitrogen-containing compounds into free amino acids. These compounds not only serve as carbon sources consumed during yeast growth but also prevent fermentation arrest (Liu et al., 2018). Crucially, this amino acid consumption aligns with their precursor roles in the Ehrlich pathway—they undergo deamination and decarboxylation to form higher alcohols, which are subsequently esterified into volatile esters (Xu et al., 2018).

Thus, the synergistic consumption of these key sugars and amino acids provides direct evidence for increased levels of fruity and floral esters (e.g., ethyl decanoate and amyl acetate) and complex alcohols (e.g., phenethyl alcohol) in wine samples, revealing a causal link between precursor utilization and flavour composition.

Among the 27 fatty acids and fatty acyls detected, 8(*R*)-hydroperoxylinoleic acid, stearidonic acid, azelaic acid, and sebacic acid levels were significantly increased.

The 20-carboxy-leukotriene B4, alpha-linolenic acid, 2-hydroxyadipic acid, and 9-oxoODE levels were significantly decreased (Fig. 2D). *S. cerevisiae* and other wine yeast strains are unable to produce fatty acids when they are grown under anaerobic winemaking conditions (Pinu et al., 2019). Thus, the fatty acid analogues in the product are due to the addition of *C. esculentus* saccharification liquid. Lipid nutrition contributes not only to cellular metabolic processes but also to the synthesis of aroma compounds. These exogenous lipids can serve as precursors for fats, waxes, and green aroma compounds (such as hexanoic acid and specific aldehydes) through yeast-mediated β-oxidation or other modifications, thereby enriching the overall aroma profile.

(A)Volcano plot for two group wines.(B)Saccharides metabolite volcano plot.

(C)Amino acids metabolite volcano plot.(D)Fatty acids and fatty acyls metabolite volcano plot.

### 3.2 Analysis of metabolic mechanisms based on KEGG pathways

**Fig. 3:**
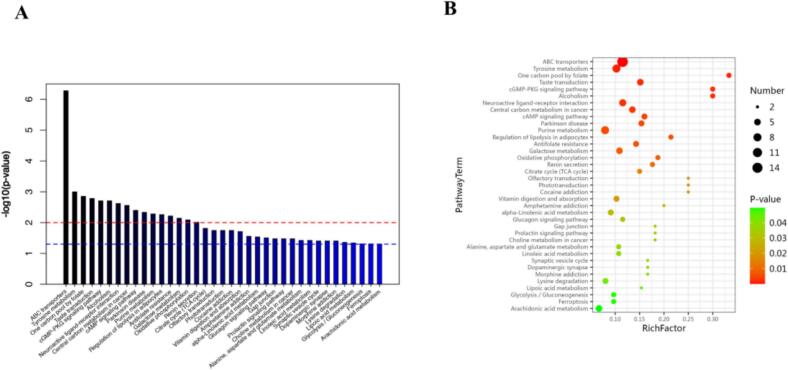
A UHPLC-OE-MS metabonomic analysis of two fruit wines.

**Fig. 4:**
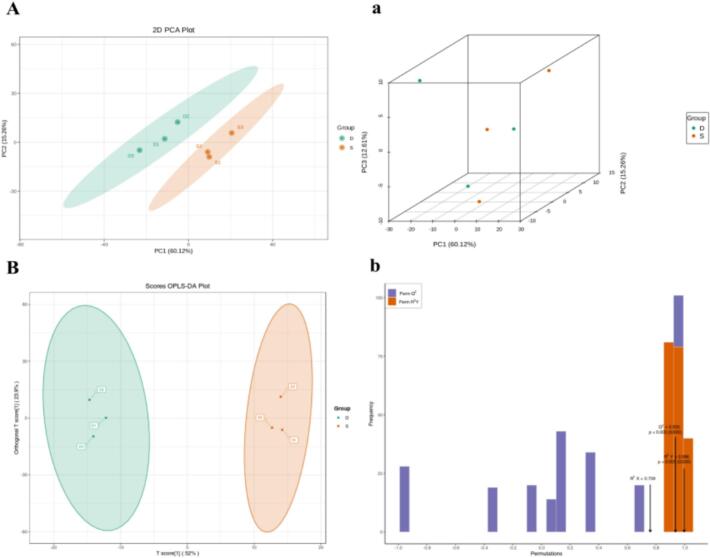
A HS-SPME-GC–MS metabonomic analysis of two fruit wines. (A) Score plot (PCA) for two group wines. (a) Score plot 3D (PCA) for two group wines. (B)OPLS-DA Score plot for two group wines. (b) OPLS-DA permutation plot for two group wines.

**Fig. 5:**
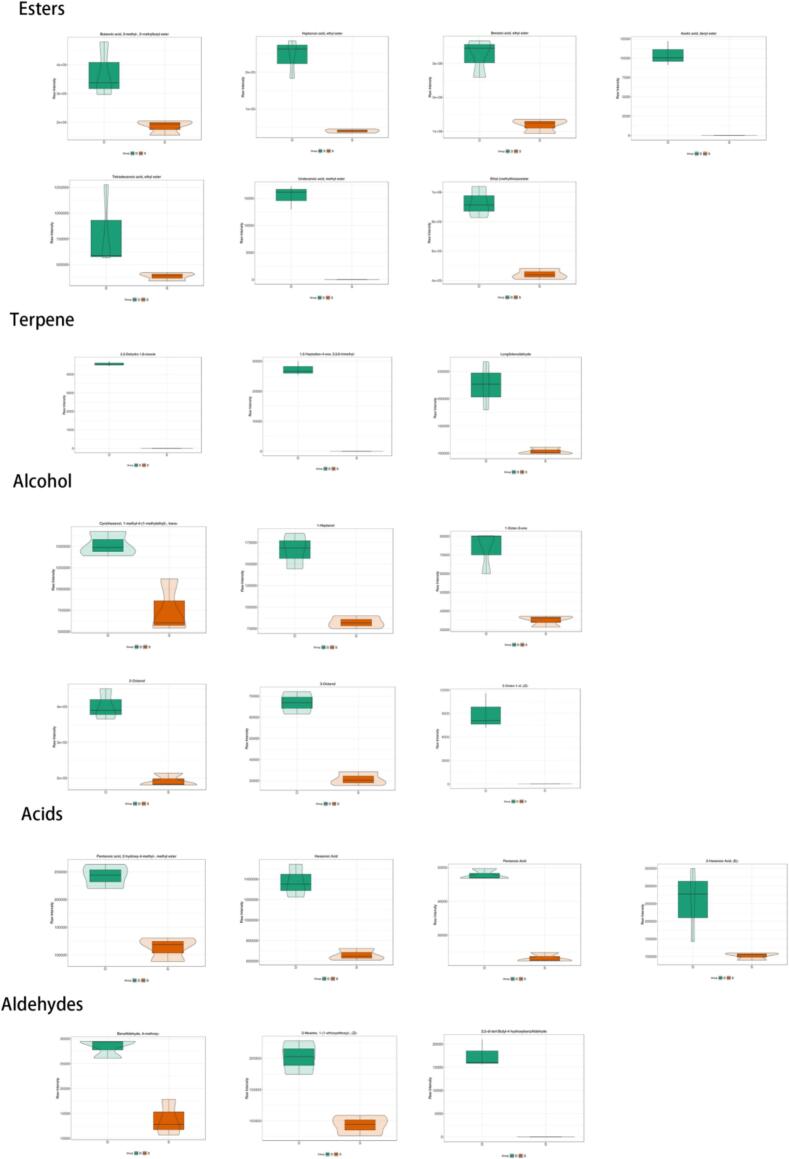
Classification of two types of wine violin chart.

KEGG pathway enrichment analysis revealed 37 significantly altered metabolic pathways (Fig. 3A and 3B). Among these, alterations in tyrosine metabolism, ABC transporters, and cAMP signalling pathways were particularly relevant to the observed conversion of flavour precursors and enhanced energy metabolism. Specifically, alterations in the cAMP signalling pathway—a central regulator of energy metabolism—suggest that it may increase glycolytic flux. This process likely provides ample carbon skeletons and energy for the Ehrlich pathway, facilitating the conversion of amino acids (such as tyrosine) into flavour compounds (e.g., thujone, which imparts hop flavour). (See Fig. 4, Fig. 5.)

(A)kegg enrichment pathway of metabolic differentials.

(B)Bubble plot of kegg enrichment pathway of metabolic differences.

#### 3.2.1 Altered metabolites underpin enhanced organoleptic and functional qualities

Compared with Group S, Group D was significantly enriched in azelaic acid, chlorogenic acid, guanosine, and D-tartaric acid (Table 2). Among these compounds, 1-pyrroline enhances aroma complexity; chlorogenic acid (Zhang et al., 2021) and guanosine (Wang et al., 2022) extend shelf life through antioxidant and colour-stabilizing effects, respectively; and tartaric acid (Liu et al., 2022) regulates acidity and inhibits contaminating microorganisms. Conversely, the levels of threonyl-aspartic acid, adenosine, 2′-deoxyadenosine, malonic acid, L-proline, and glycerophosphocholine significantly decreased in Group D (Table 3), potentially reducing allergenic risk, mitigating bitterness, and optimizing yeast carbon and nitrogen flux. The synergistic changes in these up- and downregulated metabolites simultaneously increase flavour complexity, sensory stability, and functionality in compound fruit wines while also providing precursors and reducing the capacity for subsequent volatile aroma synthesis. (See Table. 4, Table. 5.)

**Table. 2:** Statistical table of metabolite up-regulation (first 5 rows).

MS2 name	HMDB	CAS	Super.Class	Class
1-Pyrroline	HMDB0012497	5724-81-2	Organoheterocyclic compounds	Pyrrolines
Azelaic acid	HMDB0000784	123–99-9	Lipids and lipid-like molecules	Fatty Acyls
Chlorogenic acid	HMDB0003164	327–97-9	Organic oxygen compounds	Organooxygen compounds
Guanosine	HMDB0000133	118–00-3	Nucleosides, nucleotides, and analogs	Purine nucleosides
D-Tartaric acid	HMDB0029878	147–71-7	Organic oxygen compounds	Organooxygen compounds

**Table. 3:** Statistical table of metabolite downregulation (first 5 rows).

MS2 name	HMDB	CAS	Super.Class	Class
Threoninyl-Aspartate	HMDB0029057		Organic acids and derivatives	Carboxylic acids and derivatives
Adenosine	HMDB0000050	58–61-7	Nucleosides, nucleotides, and analogs	Purine nucleosides
Deoxyadenosine	HMDB0000101	958–09-8	Nucleosides, nucleotides, and analogs	Purine nucleosides
Malonic acid	HMDB0000691	141–82-2	Organic acids and derivatives	Carboxylic acids and derivatives
L-Proline	HMDB0000162	147–85-3	Organic acids and derivatives	Carboxylic acids and derivatives

**Table. 4:** Differential metabolites of compound fruit wines and raspberry wines.

Class	Compounds	D1	D2	D3	S1	S2	S3	VIP	*P*-value	Fold_Change	Log2FC
Ester	Heptanoic acid, ethyl ester	263,000.00	183,000.00	285,000.00	40,100.00	45,500.00	34,300.00	1.36	0.02	6.10	2.61
	Benzoic acid, ethyl ester	2,590,000.00	3,450,000.00	3,670,000.00	1,240,000.00	1,350,000.00	940,000.00	1.34	0.02	2.75	1.46
	Ethyl (methylthio)acetate	1,040,000.00	827,000.00	913,000.00	408,000.00	483,000.00	439,000.00	1.34	0.01	2.09	1.06
	Acetic acid, decyl ester	9120.00	10,000.00	12,200.00	0.00	0.00	0.00	1.38	0.01	1.00	1.00
	Undecanoic acid, methyl ester	17,200.00	16,200.00	13,000.00	0.00	0.00	0.00	1.38	0.01	1.00	1.00
	Pentanoic acid, 2-hydroxy-4-methyl-, methyl ester	264,000.00	220,000.00	244,000.00	88,200.00	130,000.00	119,000.00	1.32	0.00	2.16	1.11
Terpenoids	1,5-Heptadien-4-one, 3,3,6-trimethyl-	29,900.00	26,600.00	25,400.00	0.00	0.00	0.00	1.38	0.00	1.00	1.00
	Longifolenaldehyde	227,000.00	180,000.00	268,000.00	111,000.00	102,000.00	98,500.00	1.31	0.04	2.17	1.12
	Cyclohexanol, 1-methyl-4-(1-methyl ethyl)-, trans-	1,670,000.00	1,490,000.00	1,390,000.00	1,120,000.00	603,000.00	541,000.00	1.16	0.04	2.01	1.01
Alcohol	3-Octen-1-ol, (*Z*)-	8080.00	7160.00	11,500.00	0.00	0.00	0.00	1.36	0.02	1.00	1.00
	1-Heptanol	169,000.00	144,000.00	186,000.00	89,700.00	81,700.00	74,700.00	1.33	0.01	2.03	1.02
	3-Octanol	72,100.00	61,500.00	66,900.00	27,700.00	34,200.00	30,300.00	1.36	0.00	2.17	1.12
	2-Octanol	450,000.00	365,000.00	390,000.00	183,000.00	214,000.00	181,000.00	1.35	0.01	2.09	1.06
Acid	Hexanoic Acid	1,540,000.00	1,220,000.00	1,350,000.00	610,000.00	724,000.00	648,000.00	1.34	0.01	2.08	1.06
	3-Pentanoic Acid, 2,2-dimethyl-	62,000.00	52,300.00	57,300.00	23,800.00	29,900.00	25,600.00	1.35	0.00	2.16	1.11
	Pentanoic Acid	46,800.00	46,900.00	49,700.00	22,400.00	24,900.00	22,600.00	1.38	0.00	2.06	1.04
Aldehyde	3,5-di-tert-Butyl-4-hydroxybenzAldehyde	15,600.00	16,000.00	21,000.00	0.00	0.00	0.00	1.38	0.01	1.00	1.00
	BenzAldehyde, 4-methoxy-	29,400.00	26,100.00	29,500.00	17,800.00	12,800.00	10,700.00	1.27	0.01	2.06	1.04
	3-Hexene, 1-(1-ethoxyethoxy)-, (*Z*)-	203,000.00	174,000.00	228,000.00	109,000.00	94,500.00	76,300.00	1.31	0.01	2.16	1.11
Heterocyclic compound	2-Acetyl-3-methyl pyrazine	40,300.00	43,500.00	62,600.00	19,000.00	18,500.00	13,600.00	1.32	0.04	2.86	1.52
	Ethanone, 1-(3,5-dimethyl pyrazinyl)-	728,000.00	852,000.00	1,080,000.00	345,000.00	313,000.00	261,000.00	1.35	0.02	2.89	1.53
Hydrocarbons	Pentadecane, 2-methyl-	32,300.00	26,000.00	49,600.00	0.00	0.00	0.00	1.37	0.04	1.00	1.00
Phenol	Phenol, 3,5-dimethyl-	908,000.00	973,000.00	1,370,000.00	423,000.00	427,000.00	323,000.00	1.33	0.04	2.77	1.47
Amine	BenzenAmine, 2,5-dimethyl-	21,100.00	23,100.00	29,900.00	9520.00	10,600.00	8740.00	1.36	0.03	2.57	1.36
Ketone	1-Octen-3-one	80,000.00	59,900.00	80,300.00	37,200.00	36,200.00	31,400.00	1.31	0.02	2.10	1.07

**Table. 5:** Table of flavouring substances.

Compounds	Class	Odor
Heptanoic acid, ethyl ester	Ester	fruity, pineapple, cognac, rummy, wine
Benzoic acid, ethyl ester↑	Ester	fruity, dry, musty, sweet, wintergreen
Tetradecanoic acid, ethyl ester	Ester	sweet, waxy, violet, orris
Ethyl (methylthio)acetate↑	Ester	sulfury, green, fruity, tropical
1-Octen-3-one↑	Ketone	mushroom
2-Hexenoic Acid, (*E*)-	Acid	powerful, fruity, sweet, warm, herbal
Hexanoic Acid↑	Acid	rose, geranium, cheese, fatty
Pentanoic Acid	Acid	sickening, putrid, acid, sweaty, rancid
BenzAldehyde, 4-methoxy-	Aldehyde	sweet, powdery, mimosa, floral, hawthorn, balsamic
Phenol, 3,5-dimethyl-	Phenol	balsamic, coffee
3-Octanol↑	Alcohol	earthy, mushroom, herbal, melon, citrus, woody, spicy, minty
2-Octanol↑	Alcohol	fresh, spicy, green, woody, herbal, earthy
1-Heptanol	Alcohol	grassy

### 3.3 Statistical analysis of volatile compounds

Volatile aroma analyses were conducted using HS–SPME followed by GC–MS. A total of 431 volatiles were found in the fruit wine. These compounds included 83 esters, 86 terpenes, 36 alcohols, 32 aldehydes, 29 ketones, 17 acids, and others. Partial least squares discriminant analysis was used to analyse the effect of the *C. esculentus* saccharification liquid on the volatile compounds in the raspberry wine. The two groups of samples were well separated, and the high degree of aggregation of the samples within the groups indicated that there were significant differences in the volatile compounds between the two wines.

#### 3.3.1 Esters and alcohols: Direct links to sugar and amino acid metabolism

The most pronounced improvement in flavour stems from increased esters and alcohols contributing to fruity and floral notes. We detected a significant increase in 65 esters, such as ethyl decanoate (sweet, waxy aroma) and ethyl amyl acetate (banana flavour). Their formation directly correlated with the observed precursor changes: a significant reduction in fermentable sugars provided the carbon skeleton for increased alcohol synthesis via glycolysis. This is confirmed by the concurrent significant increase in 20 alcohol compounds, including phenethyl alcohol (rosy, apple-like) and 1-heptanol (Fenoll et al., 2009).

Concurrently, the decrease in amino acids indicates their role as precursors. These amino acids are degraded during fermentation (Fenoll et al., 2009) and catalyse the reaction between alcohols and acyl-CoA via alcohol acyltransferases (Xie et al., 2022; Yang et al., 2019), providing precursors for the synthesis of esters such as ethyl acetate. Similarly, the decrease in L-proline suggests its potential utilization as a nitrogen source under these fermentation conditions.

In summary, sugarcane molasses provides abundant fermentable sugars and amino acids that are efficiently utilized by yeast. This leads to enhanced metabolic flux in the esterification pathway, where alcohols (derived from sugars and amino acids) react with acyl-CoA to produce multiple esters that impart the characteristic fruity aroma to the final product.

#### 3.3.2 Terpenes and fatty acids: Contribution from raw materials and yeast modification

Profiles of terpenes and organic acids are profoundly influenced by both raw materials and yeast activity. Among the 84 terpenes detected, 42 contribute to flavour. Terpenes exist in both free volatile forms and bound glycoside forms, with the latter serving as a reservoir for aroma release through enzymatic hydrolysis during fermentation (Fenoll et al., 2009). Key terpenes include trans-β-ionone (characteristic raspberry aroma) and linalool, the latter synthesized from DHP via the isoprenoid pathway and convertible to other terpenes through various reactions (Yang et al., 2019). Studies indicate that Rubus species are rich in terpenes and diterpenes (Chen et al., 2017). The unique presence of 2,3-dihydro-1,8-eucalyptol and 1,5-heptadien-4-one, 3,3,6-trimethyl (contributing minty and lemony notes) and the disappearance of *Z*-α-trans-bergamotene demonstrate that oilseed rape addition reshapes the terpene profile, potentially through substrate competition or altered yeast enzyme activity.

The composition of organic acids also changed. Seventeen organic acids were detected, with hexanoic acid (rosy, cheesy, and fatty notes) being the most abundant. Organic acids are formed during fermentation through yeast metabolism and aldehyde oxidation (Fenoll et al., 2009). The significant upregulation of valeric acid, 2-methyl-2-pentenoic acid, and hexanoic acid in the compound wine indicates that the abundant carbon sources provided by the saccharified wort induced alterations in yeast metabolic pathways, thereby contributing to the complex acid and fatty aromas.

#### 3.3.3 Aldehydes, ketones, and functional metabolites: Enriching the flavour profile and enhancing

Flavour complexity is further enhanced by carbonyl compounds and other functional metabolites. A total of 61 aldehydes and ketones were detected, contributing to diverse sensory characteristics, such as the metallic taste of 1-hepten-3-one, the lemon and fatty notes of 2-nonenal, and the mushroom flavour of 3,5-octadien-2-one. Differences in the abundance of specific compounds, such as 1-octen-3-one, highlight the specific impact of oilseed fermentation on these secondary aroma pathways.

### 3.4 Correlation analysis between sensory characteristics and metabolites

In summary, the addition of *C. esculentus* saccharification liquid to raspberry wine followed by the addition of several aromatic compounds enriched the flavour composition of the fruit wine and enhanced the complexity and overall aroma profile of the product.

**Fig. 6:**
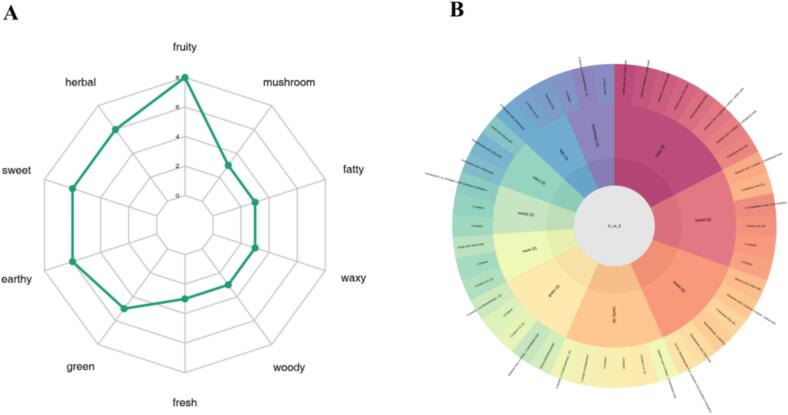
Sensory flavour radar map/Sankey map.

For the differentially abundant metabolites and organoleptic flavour profiles identified on the basis of the screening criteria in the differential comparison group, the ten most abundant organoleptic flavour compounds were selected for the generation of a radargram/sankeyMATIC flow diagram of flavouring substances.(Fig. 6).

On the basis of the flavour compounds detected in the *C. esculentus* meal raspberry compound fruit wine, the primary flavours include fruity notes, rose aroma, sweetness, green notes, honey flavour, fatty notes, nutty notes, and woody notes (Table 5). This product is a compound fruit wine characterized by a dominant raspberry fruit aroma, featuring a harmonious and rich bouquet.

## 4 Conclusion

This study successfully developed a novel composite fruit wine using hydrolysate from oilseed rape meal and red raspberries. Comprehensive metabolomics and flavour profiling revealed that oilseed rape addition significantly reshaped the metabolic profile, increasing the complexity of key flavour compounds (esters, terpenes, alcohols) and introducing unique constituents. These changes were mechanistically linked to the observed synergistic consumption of sugar and amino acid precursors, indicating enhanced flux in classical fermentation pathways.

The observed metabolic shifts align with existing biochemical knowledge. The consumption of amino acids (including tyrosine) corresponds to their role in the Ehrlich pathway, where they are converted into higher alcohols and esters—key flavour precursors essential for the development of characteristic aromas (Behringer et al., 2024, Álvarez-Fernández et al., 2019). Furthermore, the abundant carbon sources provided by oilseed rape may influence energy metabolism. cAMP, a known global regulator, promotes glycolysis through the PKA and EPAC pathways (Kilanowska & Ziółkowska, 2020), while a “glycolysis-enhanced” metabolic state can supply excess carbon skeletons and abundant ATP energy to the Ehrlich pathway (Yu et al., 2022). Thus, the introduction of oilseed rape likely enhances the aroma of fruit wine by providing abundant substrates to pathways such as the Ehrlich pathway and potentially creating an energy environment favourable for flavour biosynthesis. Although precise regulatory mechanisms require further validation, the observed metabolic shifts align with a hypothetical scenario in which enhanced precursor availability drives desirable flavour compound formation while reducing undesirable flavour compounds. The potential role of global regulators, such as the cAMP signalling pathway, in coordinating this metabolic reprogramming warrants further investigation in future studies.

## 5 Future perspectives

Given that this study employed the HS–SPME–GC–MS single-point internal standard method, the absence of pure standard curves and response factor corrections for all target volatiles precludes the calculation of absolute concentrations required for determining OAVs. Therefore, OAV data are not provided here. Instead, compounds detected at relatively high concentrations (marked with “↑” in Table 5) are listed as “potential aroma contributors” on the basis solely of olfactory thresholds reported in the literature. Subsequent work will employ stable isotope dilution coupled with a multipoint calibration curve method to achieve absolute quantification and systematically calculate the OAV.

## CRediT authorship contribution statement

**Chang Yu:** Writing – review & editing, Writing – original draft. **Yang Liu:** Methodology, Data curation. **Yintu Na:** Writing – original draft. **Xiaotong Wu:** Supervision.

## Declaration of competing interest

The authors declare that they have no knowncompeting financial interests or personarelationships that could have appeared to influence the work reported in this paper.

## Data Availability

Data will be made available on request.
